# Role of m6A Methylation Regulators in the Diagnosis and Subtype Classification of COPD Based on the GEO Database

**DOI:** 10.1111/jcmm.70226

**Published:** 2024-11-24

**Authors:** Pingan Zhang, Na Gao, Xiaoning Li, Xudong Zheng, Deyu Kong, Jianjun Wu

**Affiliations:** ^1^ Respiratory Department, the Third Affiliated Hospital Beijing University of Chinese Medicine Beijing China; ^2^ Rehabilitation College Zhengzhou Health Vocational College Zhengzhou China

**Keywords:** COPD, diagnosis, differential genes, m6A methylation regulators, subtype

## Abstract

N6‐methyladenosine (m6A) is a prevalent mRNA modifier, yet its role in chronic obstructive pulmonary disease (COPD) remains unexplored. We sourced expression levels of m6A methylation regulators from the GSE76925 dataset. These regulators' differential expression (DEMs) predicted COPD risk via random forest and support vector machine models. Additionally, a nomogram model using DEMs estimated COPD prevalence. We employed consistent cluster analysis of m6A methylation regulators to categorise COPD samples into distinct subtypes. Analyses of immune cell infiltration in these subtypes and differential gene expression (DEGs) across m6A methylation subtypes were conducted. A cell model validated several m6A regulators and their associated pathways. Fifteen m6A methylation regulators showed differential expression and were used in random forest and support vector machine models. Eleven were selected for a nomogram model, which decision curve analysis suggested could benefit patients. Consensus cluster analysis divided the COPD samples into two subtypes: Cluster A and Cluster B. Cluster B was associated with neutrophil and eosinophil‐dominated immunity, while Cluster A was linked with monocyte‐dominated immunity. Validation of some research findings was achieved through cell experiments. m6A methylation regulators appear instrumental in diagnosing and classifying subtypes of COPD.

## 1 Introduction

Chronic obstructive pulmonary disease (COPD) is a treatable and preventable condition characterised by a progressive decline in lung function. It manifests with persistent respiratory symptoms and irreversible airflow restriction due to small airway obstruction [1]. The pathogenesis of COPD involves neutrophils, eosinophils and oligogranulocytes, which can trigger inflammatory reactions leading to emphysema, ciliary dysfunction and other vital pathological features [2]. Research indicates significant recruitment of macrophages and neutrophils to the airways in COPD patients, alongside an increase in T lymphocytes and B lymphocytes within the airway walls, underlining the potential of genome editing in identifying COPD‐related genes and biomarkers for therapeutic strategies [3].

Over 100 different RNA modifiers, including N6‐methyladenosine (m6A), 5‐methylcytidine (m5C) and N1‐methyladenosine, have been identified in eukaryotic cells [4]. Previous studies have examined the role of m5c methylation regulators in COPD [4]; however, the function of m6A methylation regulators remains unclear. First identified in 1974, m6A methylation regulators modulate several biological processes [5]. These regulators include adenosine methyltransferases (writers), demethylases (erasers) and binding proteins (readers) [6]. Notably, m6A writers such as WT1‐associated protein (*WTAP*), methyltransferase 3/14 (*MEETL3/14*), RNA binding protein 15/15b (*RBM15/15B*), zinc finger protein 217 (*ZNF217*) and Cbl proto‐oncogene‐like 1 (*CBLL1*) [7, 8] and erasers such as obesity‐associated protein (*FTO*) and fat block alkB homology 5 (*ALKBH5*) [9, 10] have been catalogued. Readers include yt521‐b homology (*YTH*) domain‐containing proteins (*YTHDC1/2*), insulin‐like growth factor 2 mRNA binding proteins (*IGF2BP1/2/3*) and heterogeneous nuclear ribonucleoproteins (*HNRNPA2B1/HNRNPC*) [11, 12, 13]. These m6A regulators influence cell differentiation, the cell cycle, circadian rhythms and human diseases by modulating mRNA structure, maturation, splicing, translation, localisation and decay [14]. Furthermore, emerging evidence suggests that m6A methylation regulators are pivotal in various diseases, the tumour microenvironment, immune modulation and inflammatory factor release [15, 16]. Research has highlighted that several m6A methylation regulatory factors, such as METTL3, METTL14, FTO and YTHDF1, are implicated in the onset and progression of COPD [17, 18, 19, 20, 21]. Specifically, METTL3 expression is significantly elevated in COPD patients, smoke‐induced COPD animal models and cell models [18, 20]. METTL3‐mediated m6A RNA methylation regulates CSE‐induced epithelial–mesenchymal transition by targeting SOCS3 mRNA, thereby playing a crucial role in COPD development [18, 19]. However, the underlying regulatory mechanisms of m6A methylation regulators in COPD remain to be elucidated.

This study employs the GSE76925 dataset from the GEO database to investigate the diagnostic and subtype‐related roles of m6A in COPD.

## 2 Materials and Methods

### 2.1 Data Acquisition

The GSE76925 dataset from the GEO database, encompassing gene expression data from 112 COPD patients and 40 normal controls, was utilised in this study. Differential m6A methylation regulators (DEMs) between COPD and normal controls were identified using the limma package in R software. Visualisation of the chromosome positions of these m6A methylation regulators was achieved through the ‘rcircos’ package in R.

### 2.2 Constructing a Linear Regression Model to Analyse the Correlation of the Differential m6A Methylation Regulators

The correlation between ‘reader’, ‘writer’ and ‘eraser’ was analysed by the linear regression equation. Statistical significance was defined as a *p* < 0.05, in which *R* < 0 indicated a negative correlation of the data, and *R* > 0 indicated a positive correlation.

### 2.3 Establishing the Support Vector Machine and Random Forest Model

The occurrence of COPD was predicted using support vector machine (SVM) and random forest (RF) models. These models were implemented with the ‘caret’ and ‘randomForest’ packages in R, employing techniques such as uneven reverse cumulative distribution, residual boxplots and ‘roc’ curves. The DEMs were further refined through 10‐fold cross‐validation.

### 2.4 Constructing the Nomogram Model

Based on the DEMs, this model was constructed using the ‘rms’ and ‘rmda’ packages in R. This model aimed to predict the prevalence of COPD, with its accuracy assessed by a calibration curve. Decision curve analysis (DCA) and clinical impact curves were used to evaluate the model's utility for COPD patients.

### 2.5 Construction of m6A Methylation Subtypes

The ‘consensus cluster plus’ package in R software was used to analyse the DEMs regulators, and different m6A subtypes were obtained. The ‘pheatmap’, ‘reshape2’ and ‘ggpubr’ packages facilitated the creation of heatmaps and box diagrams for these subtypes. Principal component analysis (PCA) of COPD samples, conducted with the ‘limma’ and ‘ggplot2’ packages, quantified the distinct m6A methylation subtypes.

### 2.6 Evaluation of Immune Cell Infiltration in m6A Methylation Subtypes

The ‘GSEABase’ and ‘GSVA’ packages in R were employed for single‐sample gene set enrichment analysis (ssGSEA) to assess the abundance of immune cells in the m6A methylation subtypes. This analysis sorted the gene expression levels of the samples, quantifying them to ascertain the abundance of immune cells based on gene expression levels.

### 2.7 GO and KEGG Enrichment Analysis of DEGs Related to m6A Methylation Subtypes

The ‘limma’ package in R software was utilised to identify differentially expressed genes (DEGs) of m6A methylation subtypes, adhering to the criteria of logFC > 1 and an adjusted *p* < 0.05. Subsequently, gene ontology (GO) functions and Kyoto Encyclopedia of Genes and Genomes (KEGG) pathway analyses were conducted on these DEGs. The results were visualised with a GO circle diagram, GO and KEGG bubble diagrams and histograms, maintaining a significance threshold of *p* < 0.05.

### 2.8 Identification of m6A Gene Subtypes

The relevance of m6A methylation subtypes was confirmed by constructing COPD gene subtypes using the ‘ConsensusClusterPlus’ package in R. Expression heatmaps of DEGs across different m6A gene subtypes were generated. The correlation between various immune cells, m6A gene subtypes and m6A methylation subtypes was analysed using the ‘limma’ and ‘ggpubr’ packages. Additionally, the m6A score for each sample was calculated using the PCA algorithm, and the stability of the m6A methylation and gene subtypes was assessed by comparing these m6A scores.

### 2.9 Relationship Between m6A Methylation Subtypes, m6A Genes Subtype and HDAC Family Protein Expression

Histone deacetylase (*HDAC*), a critical regulator of histone acetylation and deacetylation, influences gene transcription and modulates the inflammatory response, playing a significant role in the pathophysiology of COPD [22]. To explore the involvement of the *HDAC* family in m6A methylation in COPD, the correlation between m6A methylation subtypes, m6A gene subtypes and the protein expression levels of HDAC1, HDAC2, HDAC3, HDAC4 and HDAC6 was analysed using the ‘limma’ and ‘ggpubr’ packages. The outcomes are depicted in a box diagram.

### 2.10 Experimental Validation

METTL3, NF‐κB and HDAC2 were randomly selected for validation using a COPD cell model.

### 2.11 Cigarette Smoke Extract (CSE) Preparation and CSE Concentration Screening

CSE was prepared from Daqianmen cigarettes (Shanghai Tobacco Co. Ltd). Initially, a cigarette was lit, and the smoke, totalling 300 mL, was collected and injected into 25 mL of DMEM high‐sugar medium, housed within a closed container. The pH of the culture medium was adjusted to 7.4 using 1 mol/L NaOH. Bacterial contamination was eliminated using a 0.22‐μm filter. Finally, the appropriate CSE concentration was determined using a CCK8 assay.

### 2.12 Experimental Grouping

The BEAS‐2B human bronchial epithelial cells (Catalogue No. BNCC359274, Beiner Biological Co. Ltd) were categorised into model and control groups. The model group underwent a 5% CSE intervention for 24 h, while the control group received no CSE treatment.

### 2.13 Immunofluorescence Staining of HDAC2

The cells were fixed in 4% paraformaldehyde for 20 min, treated with 0.5% Triton X‐100 for cell membrane permeabilisation for another 20 min and then blocked with goat serum. The cells were incubated overnight at 4°C with HDAC2 primary antibody (Catalogue No. 12922‐3‐AP, Proteintech) diluted 1:200. After washing with PBST (PBS + Tween‐20 lotion, pH = 7.4), the cells were incubated with a fluorescent secondary antibody in the dark for 1 h, and with DAPI for 10 min in the dark. The stained cells were observed under a fluorescence microscope.

### 2.14 Western Blot of METTL3, NF‐kB and HDAC2

For protein extraction, 100 μL of RIPA lysate supplemented with 1 μL of protease inhibitor was added to each well of a six‐well cell culture plate, incubated on ice for 20 min and then centrifuged at 4°C for 12,000 rpm. The supernatant containing the proteins was then quantified. A 5× loading buffer was added, mixed thoroughly, heated in a water bath at 100°C and stored at −80°C. The proteins were separated by SDS‐PAGE under a constant voltage of 90 V and then transferred to a membrane at 200 mA constant current. The membrane was blocked with 5% skim milk and then incubated overnight at 4°C with primary antibodies against NF‐kB (Catalogue No. 66535–1, 1:1000 dilution, Proteintech), METTL3 (Catalogue No. 67733–1, 1:1000 dilution, Proteintech), HDAC2 (Catalogue No. 12922‐3‐AP, 1:1000, Proteintech), TBP (internal control; Catalogue No. 22006‐1‐AP, 1:1000, Proteintech) and GAPDH (internal control; Catalogue No. 60004–1, 1:8000, Proteintech). After washing with TBST, the membrane was incubated with a 1:10000 diluted secondary antibody for 1 h, followed by further washes and development using enhanced chemiluminescence (ECL).

## 3 Results

### 3.1 Distribution of m6A Methylation Regulators in COPD Patients

**FIGURE 1:**
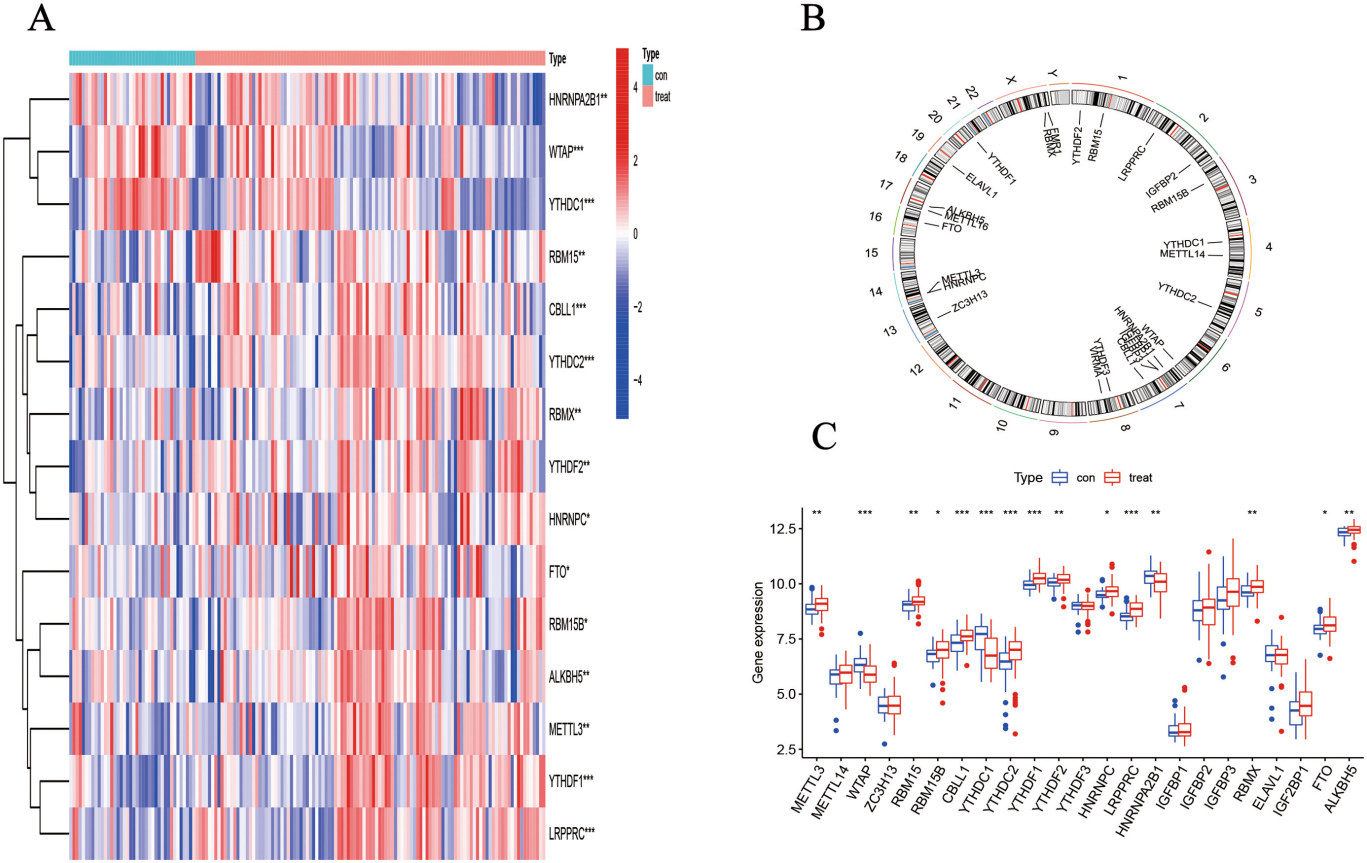
Distribution of m6A methylation regulators in COPD. (A) Heatmap of DEMs. (B) Chromosome location of m6A methylation regulators. (C) Histogram of m6A methylation regulators. Con, control group; Treat, COPD group; *p < 0.05, **p < 0.01, ***p < 0.001.

A total of 23 m6A methylation regulators were identified, including 7 ‘writers’ (*METTL3*, *METTL4*, *WTAP*, *ZC3H13*, *RBM15*, *RNM15B* and *CBLL1*), 14 ‘readers’ (*YTHDC1*, *YTHDC2*, *YTHDF1*, *YTHDF2*, *YTHDF3*, *HNNRPC*, *LPPPRC*, *HNRNPA2B1*, *IGFBP1*, *IGFBP2*, *IGFBP3*, *RBMX*, *ELAVL1* and *IGF2BP1*) and 2 ‘erasers’ (*FTO* and *ALKBH5*). Fifteen m6A methylation regulators were found to be differentially expressed (five writers: METTL3, WTAP, RBM15, RBM15B and CBLL1; eight readers: YTHDC1, YTHDC2, YTHDF1, YTHDF2, HNRNPC, LRPPRC, HNRNPA2B1 and RBMX and two erasers: FTO and ALKBH5, *p* < 0.05). The expression patterns were visually represented in heatmaps, histograms and box diagrams, revealing that many methylation regulators, including *METTL3*, *RBM15*, *RNM15B*, *CBLL1*, *YTHDC2*, *YTHDF1*, *YTHDF2*, *HNNRPC*, *LPPPRC*, *RBMX*, *FTO* and *ALKBH5*, were significantly overexpressed in COPD patients, whereas WTAP, YTHDC1 and HNRNPA2B1 showed reduced expression levels in these patients (Figure 1A–C).

### 3.2 Correlation Results of m6A Methylation Regulators in COPD Patients

**FIGURE 2:**
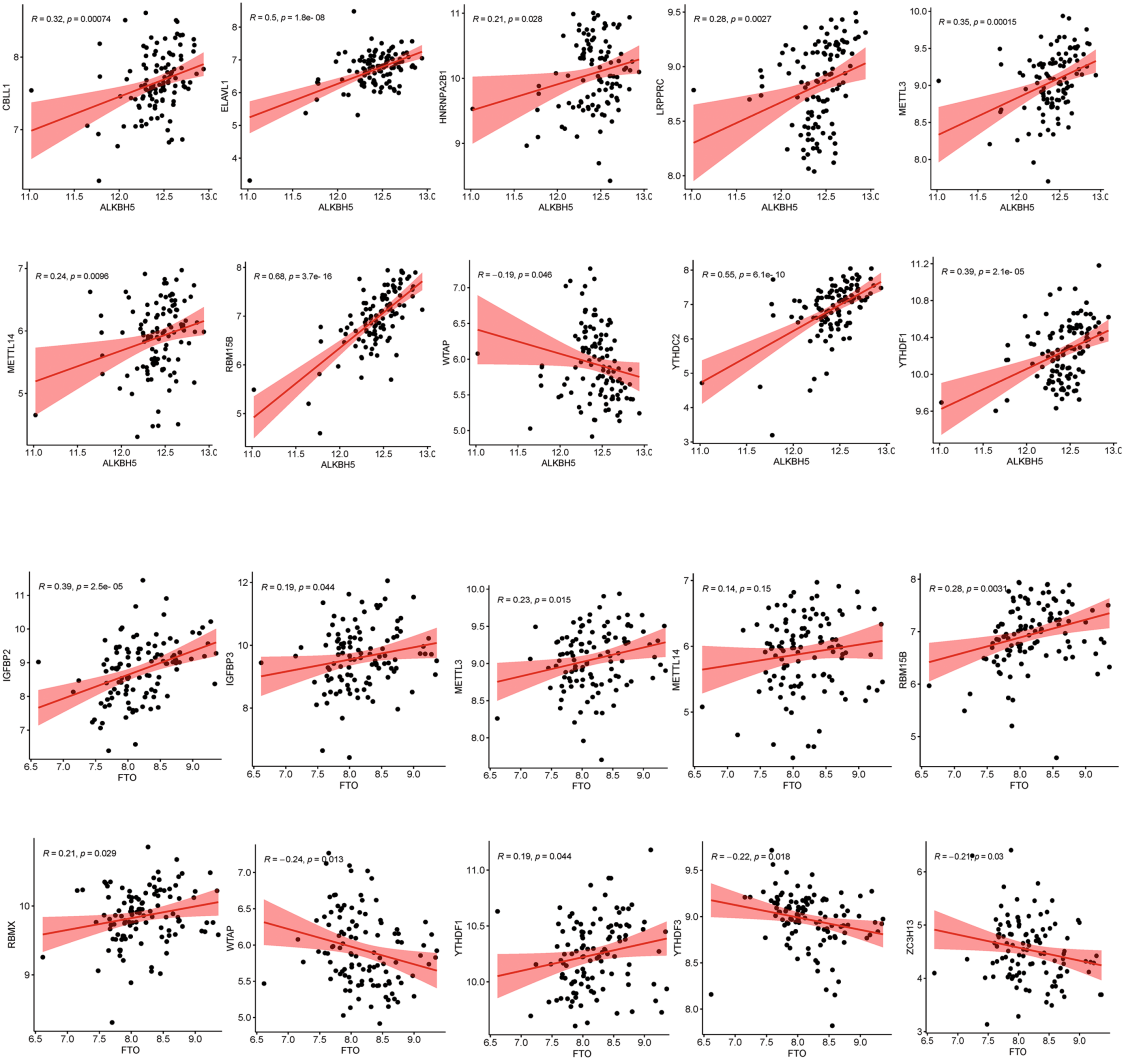
Correlation of ‘writer’, ‘reader writer’ and ‘eraser’ in m6A methylation regulators in COPD patients.

Significant correlations were observed between the categories of ‘writer’, ‘readerwriter’ and ‘eraser’. Specifically, *CBLL1*, *ELAV1*, *HNRNP2B1*, *LRPPRC*, *METTL3*, *METTL4*, *RNM15B*, *YTHDC2* and *YTHDF* showed positive correlations with *ALKB5*. Conversely, *WTAP* exhibited a significant negative correlation with *ALKB5*. Additionally, *IGFBP1*, *IGFBP3*, *METTL3*, *METTL4*, *RBM15B*, *RNMX* and *YTHDF1* were positively correlated with *FTO*, while *WTAP*, *YTHDF3* and *ZC3H13* were negatively correlated with *FTO* (Figure 2).

### 3.3 RF Model and SVM Model Results

**FIGURE 3:**
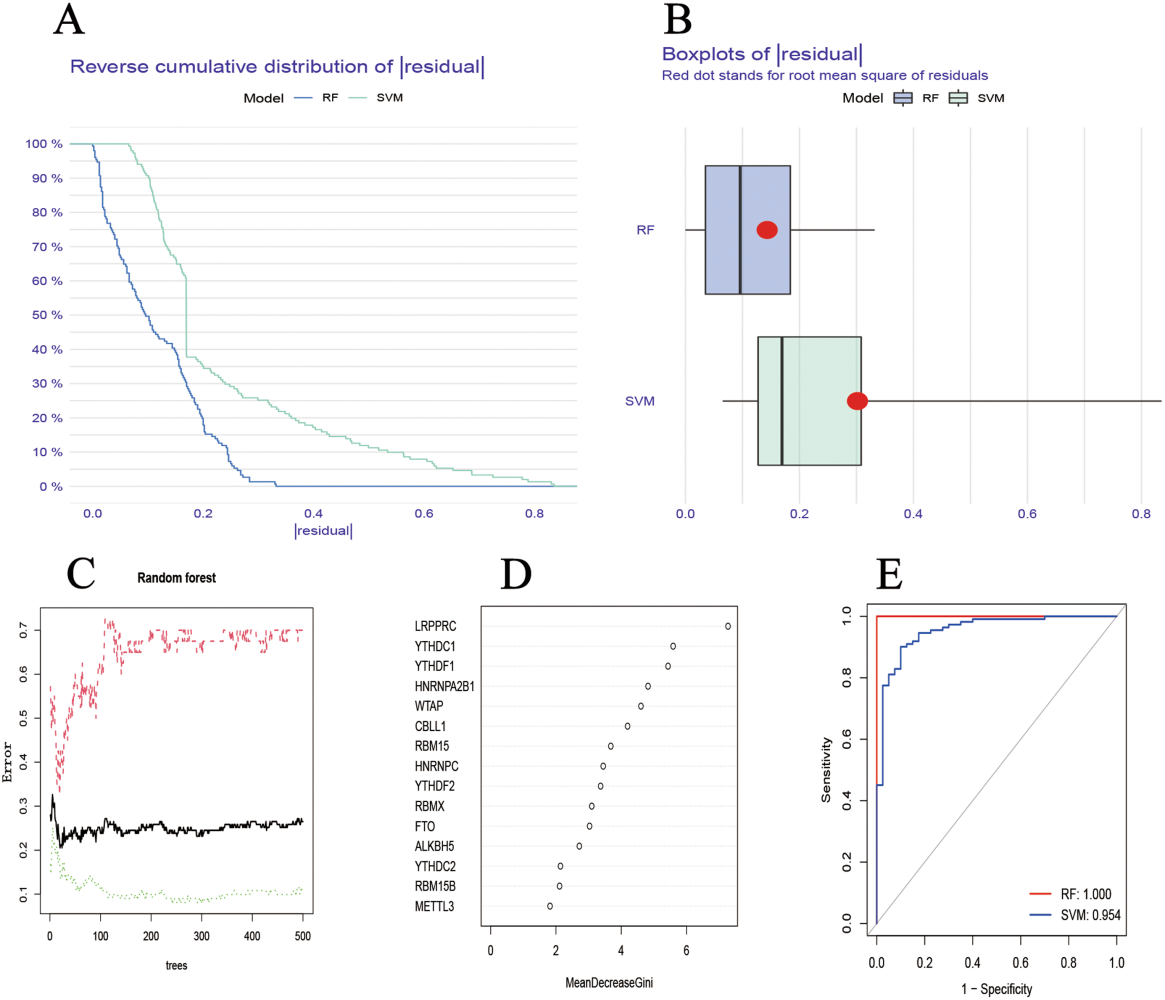
Construction of RF and SVM model. (A) The inverse cumulative distribution of residuals is plotted to show RF and SVM. (B) A residual diagram shows the residual distribution of RF and SVM. (C) Random forest tree model validation. (D) DEMs based on RF model. (E) ROC curve shows the accuracy of RF and SVM models.

The DEMs were used to predict the incidence rate of COPD through both the RF and SVM models. The ‘Reverse cumulative distribution of residuals’ (Figure 3A) and ‘boxplots of residuals’ (Figure 3B) indicated that the RF model exhibited smaller residuals compared to the SVM model, suggesting a higher stability in predicting the incidence rate of COPD. Optimal tree counts were selected based on error values across different sample groups in the RF model (Figure 3C). Genes with an importance score greater than 1 were selected, and their importance was visualised (Figure 3D). The ROC curve further validated the superior stability of the RF model, as evidenced by its larger area under the curve compared to the SVM model (Figure 3E).

### 3.4 Nomogram Model

**FIGURE 4:**
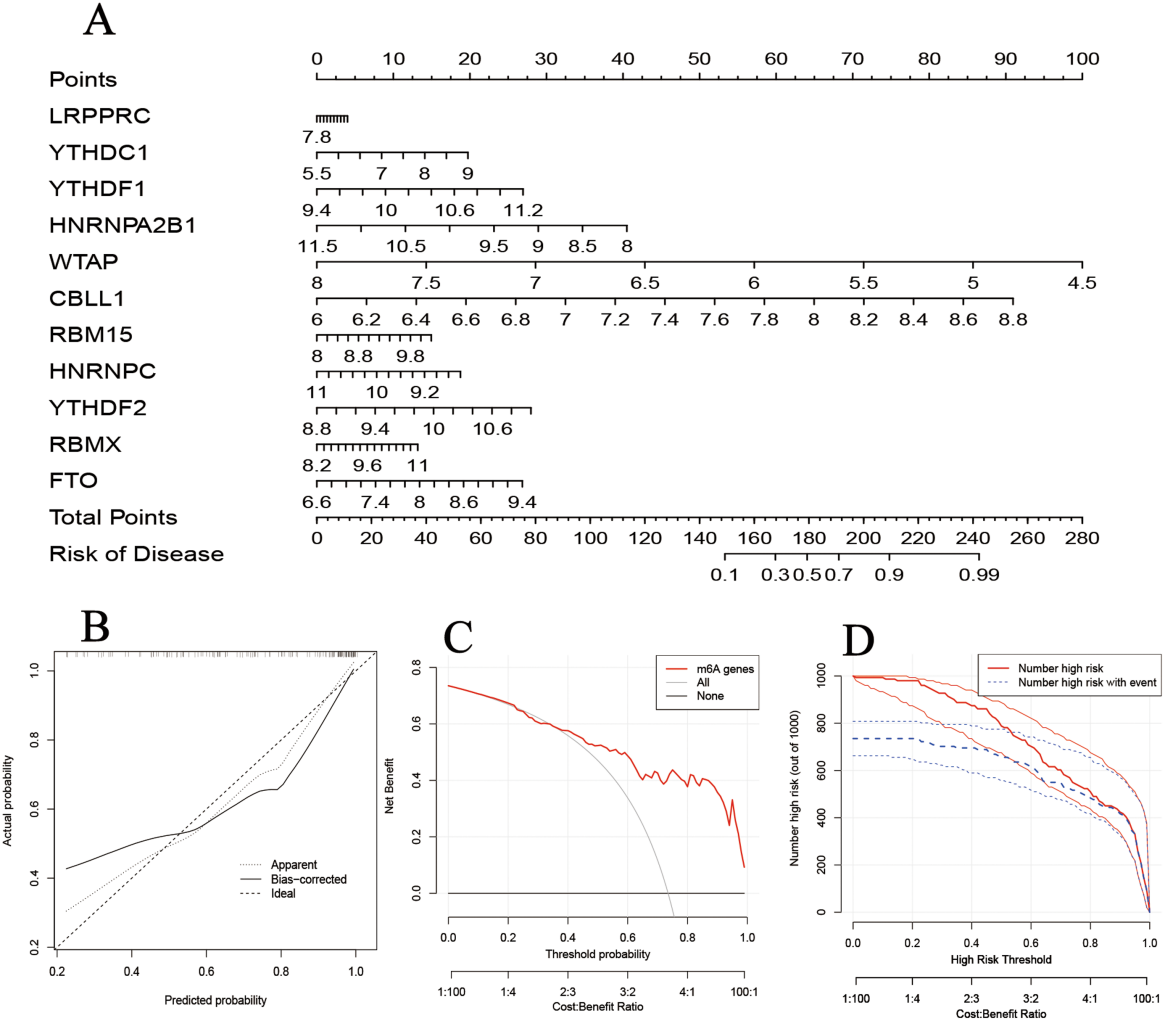
Nomogram model. (A) Construction of nomogram model based on 11 DEMs. (B) The calibration curve showed the predictive ability of the nomogram model. (C) The decision based on the nomogram model benefited COPD patients. (D) The clinical ROC curve evaluated the clinical impact of the nomogram model.

A nomogram model constructed with 11 DEMs proved stable in predicting the incidence rate of COPD (Figure 4A). The calibration curve affirmed the model's accuracy in reflecting the predicted against actual probabilities, with adjustments for bias (Figure 4B). DCA demonstrated that the model provided net benefits to COPD patients, indicating a favourable cost–benefit ratio and threshold probability (Figure 4C). The clinical prediction model underscored the efficacy of the nomogram in forecasting COPD occurrence (Figure 4D).

### 3.5 m6A Methylation Subtypes

**FIGURE 5:**
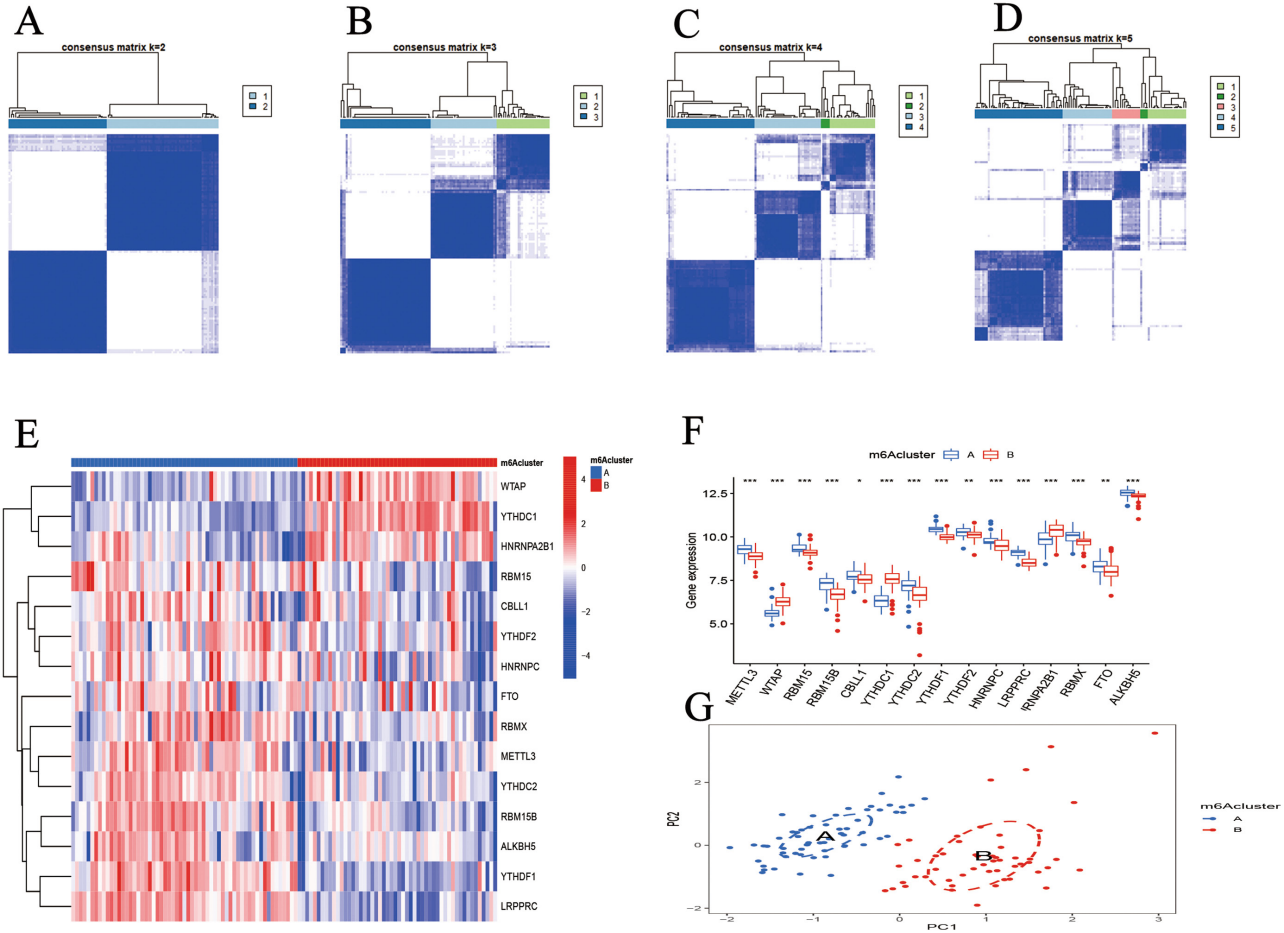
m6A methylation subtypes. (A–D) Consensus clustering of DEMs with k = 2–5. (E) Expression heat map of DEMs in cluster A and cluster B. (F) Histogram of differential expression of DEMs in cluster A and cluster B. (G) PCA of COPD patients. *p < 0.05, **p < 0.01, ***p < 0.001.

COPD patients were classified into distinct m6A methylation subtypes based on 15 DEMs, resulting in two main subtypes: Cluster A, consisting of 59 COPD patients, and Cluster B, comprising 52 patients (Figure 5A–D). Heat maps and block diagrams depicted the differential expression of DEMs across these subtypes. Cluster A prominently expressed *METTL3*, *RBM15*, *RBM15B*, *CBLL1*, *YTHDC2*, *YTHDF1*, *YTHDF2*, *HNRNPC*, *LRPPRC*, *RBMX*, *FTO* and *ALKBH5*, whereas Cluster B was characterised by higher expression levels of *WTAP*, *YTHDC1* and *HNRNPA2B1* (Figure 5E,F). PCA conclusively differentiated the COPD patients into these two subtypes using the 15 DEMs (Figure 5G).

### 3.6 Immune Cell Infiltration Results

**FIGURE 6:**
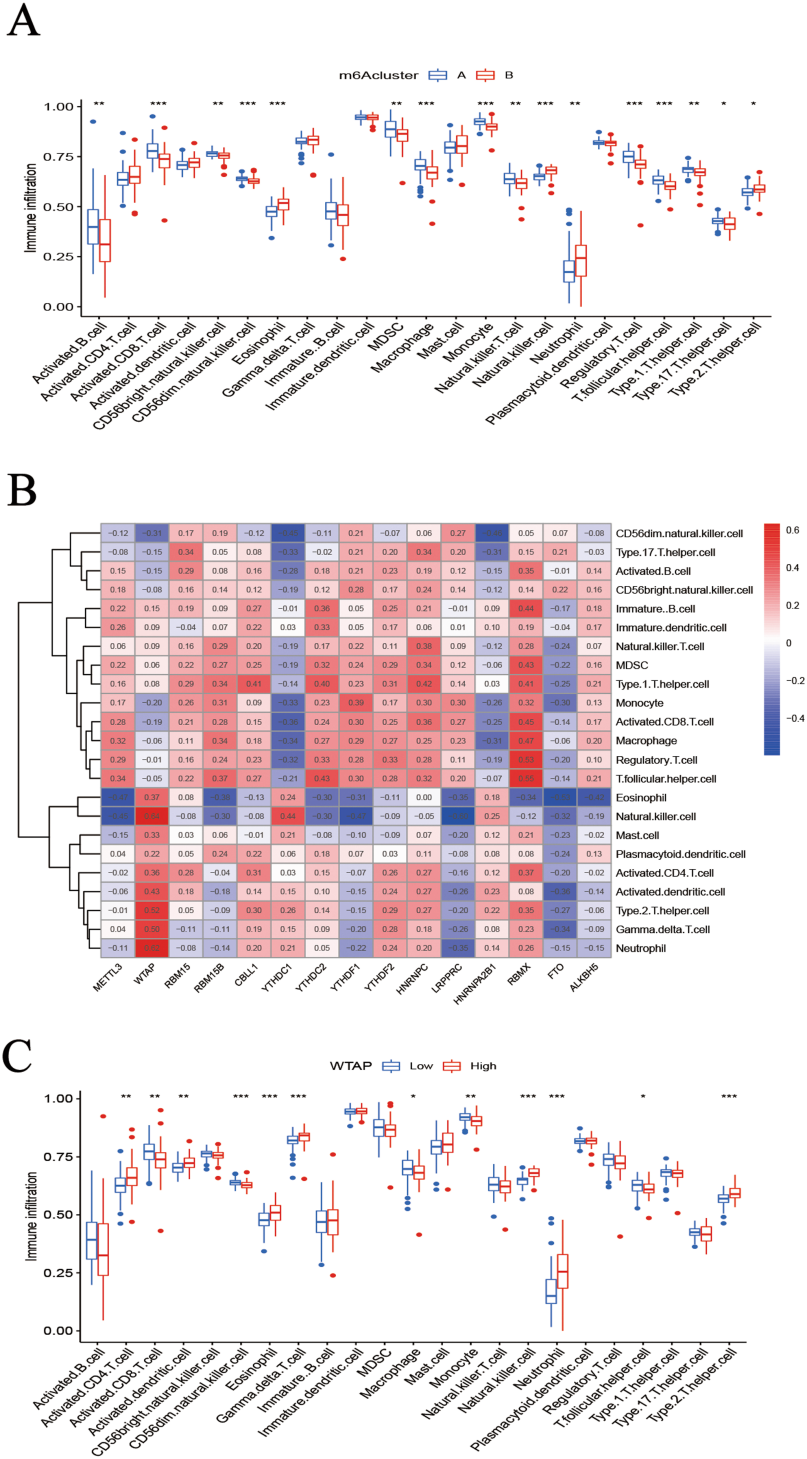
ssGSEA (A) Immune cell infiltration between Cluster A and Cluster B. (B) Correlation between the expression of DEMs and immune cells. (C) Differences in the abundance of immune cell infiltration between patients with high and low WTAP protein expression. *p < 0.05, **p < 0.01, ***p < 0.001.

The abundance of immune cells in COPD patients was determined via ssGSEA. The correlations between m6A methylation regulators and immune cells were assessed. Analysis of the two m6A subtypes and the associated box diagrams of immune cell infiltration revealed that eosinophils and neutrophils were highly enriched in Cluster B, whereas monocytes and activated B cells predominated in Cluster A (Figure 6A). This finding suggests that distinct types of cellular immunity are implicated in regulating m6A methylation in COPD. Further analysis of the heatmap detailing the correlation between m6A methylation regulators and immune cells (Figure 6B) indicated that the m6A methylation regulator *WTAP* was positively correlated with numerous immune cells. Specifically, overexpression of *WTAP* was associated with a significant positive correlation with neutrophils and eosinophils compared to the low‐expression group (Figure 6C).

### 3.7 GO and KEGG Enrichment Analysis

**FIGURE 7:**
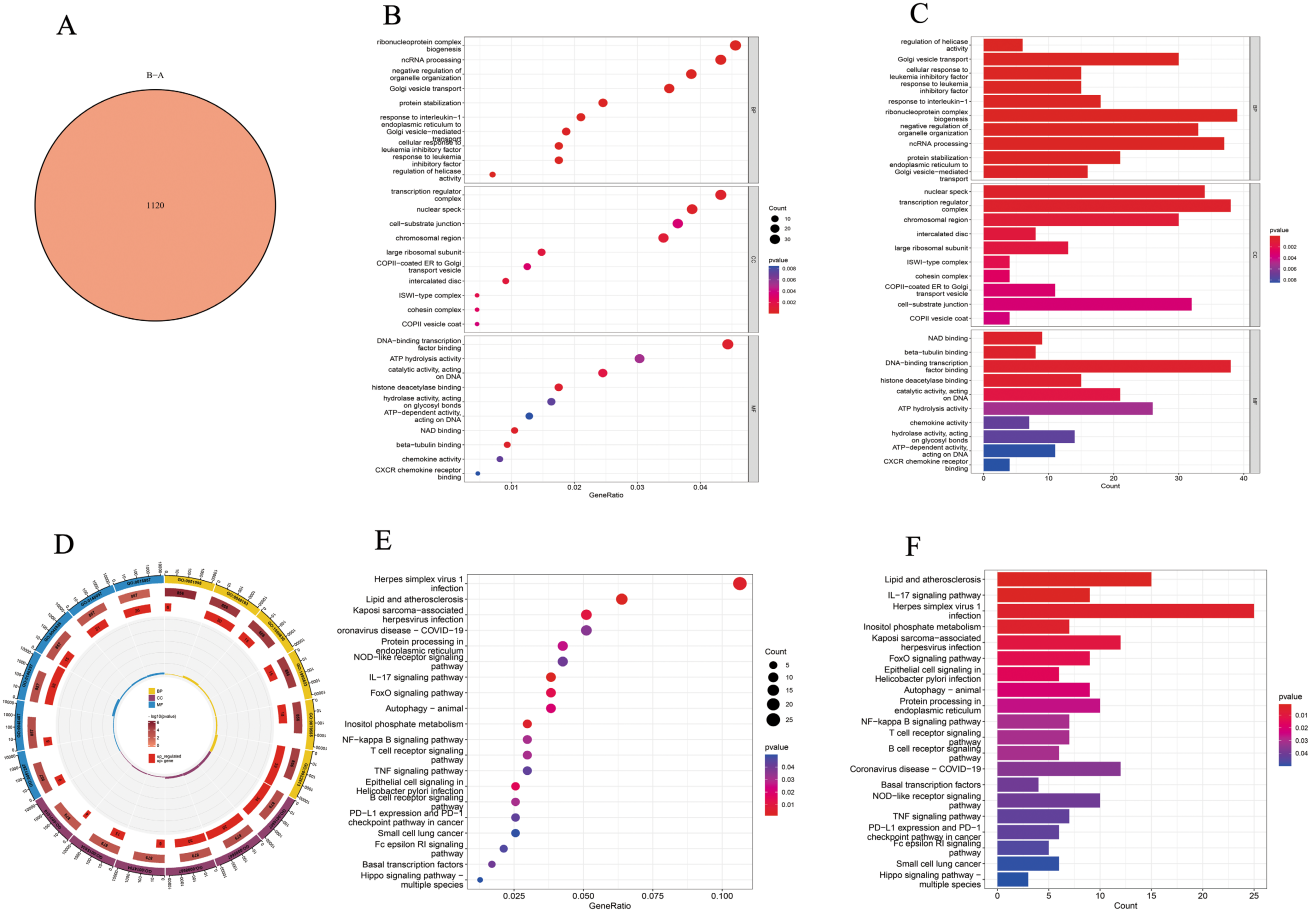
Go and KEGG of DEGs. (A) V enn diagram (B) Bubble Diagram of GO (C) histogram of GO (D) circle map of GO (E) Bubble Diagram of KEGG (F) histogram of KEGG.

A total of 1120 genes (DEGs) were identified (Figure 7A). GO analysis revealed significant enrichment in biological processes, including helicase activity regulation, response to interleukin‐1, histone deacetylase binding and CXCR chemokine receptor binding (Figure 7B–D). Concurrently, KEGG pathway analysis highlighted enrichment in pathways such as the NF‐κB signalling pathway, tumour necrosis factor signalling pathway and IL‐17 signalling pathway (Figure 7E,F).

### 3.8 Identification of Two m6A Genotypes

**FIGURE 8:**
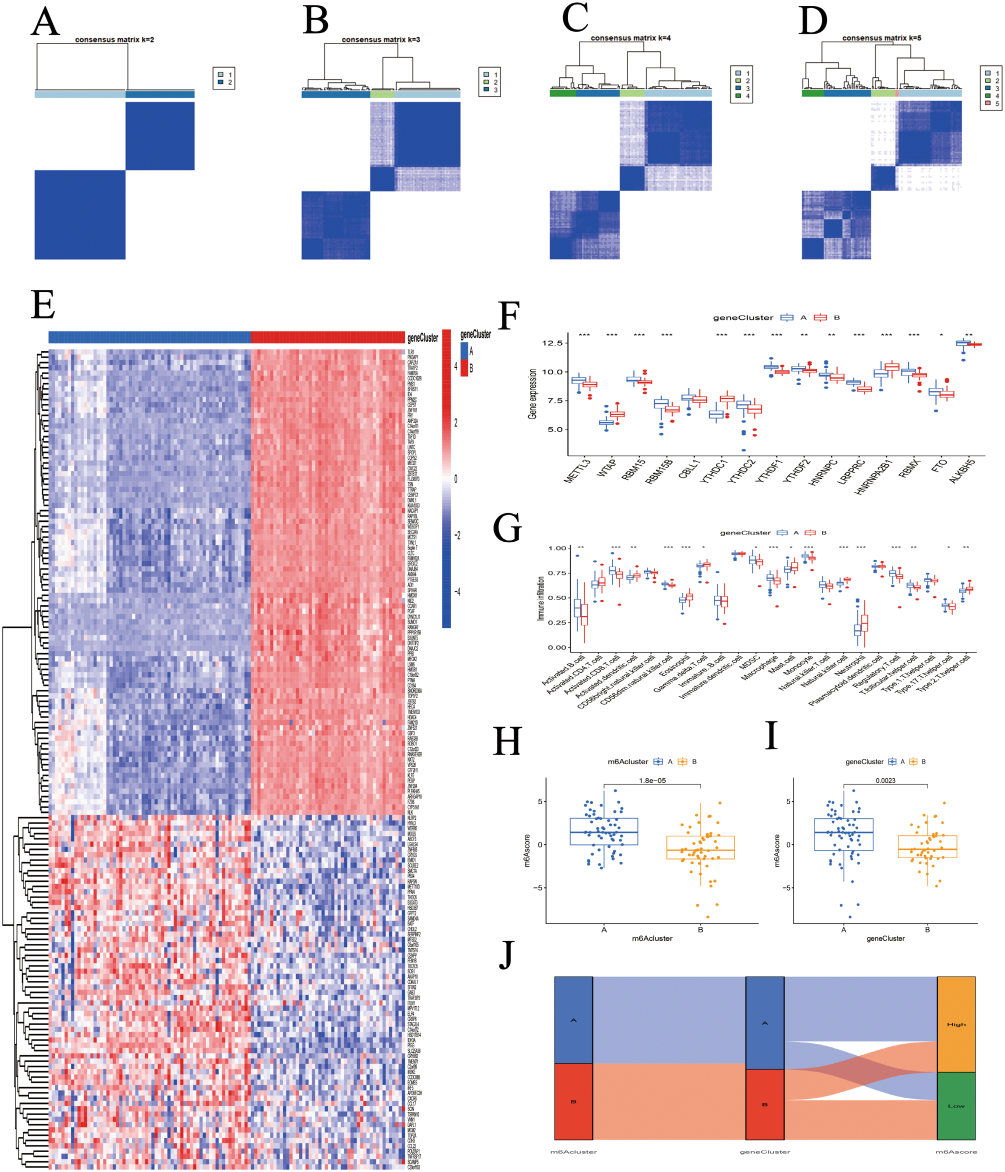
m6A genotypes. (A–D) consensus matrix of m6A genotype by DEGs with k = 2–5. (E) heatmap of DEGs related to m6A genotypes A and B. (F) Box diagram of DEMs in m6A genotype A and B. (G) The difference of immune cell infiltration between m6A genotypes. (H) m6A score in m6A methylation subtypes. (H, I) m6A score in m6A genotype. (J) Sankey diagram. *p < 0.05, **p < 0.01, ***p < 0.001.

Genotypes A and B corresponded with the m6A methylation subtypes observed in COPD patients (Figure 8A–D). The top 155 DEGs between genotypes A and B were utilised to create a gene expression heatmap (Figure 8E). A comparison of the differential expression levels of the 15 DEMs and the patterns of immune cell infiltration in m6A genotypes A and B showed similarities to the m6A methylation subtypes (Figure 8F,G). Further analysis comparing m6A scores across the different m6A methylation subtypes and m6A genotypes revealed that scores for m6A methylation subtype A and m6A genotype A were higher than those for subtype B and genotype B (Figure 8H,I). The relationships among genotypes, methylation subtypes and scores were succinctly visualised using a Sankey diagram (Figure 8J).

### 3.9 m6A Methylation Subtypes, m6A Genotype and HDAC Family Protein Expression Results

**FIGURE 9:**
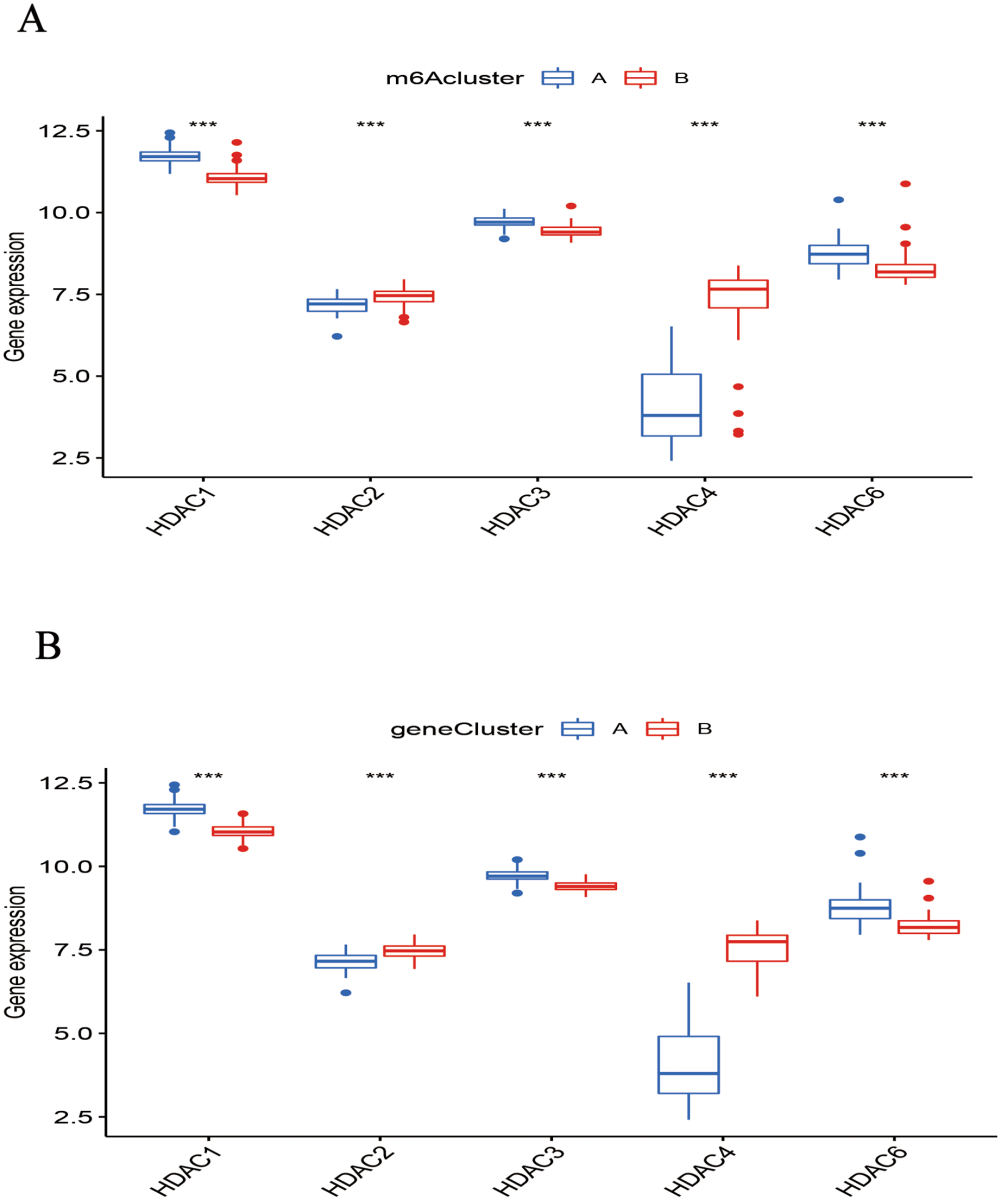
Relationships between histone acetylation family proteins, m6A methylation subtypes and m6A genotype. (A) *HDAC2* and *HDAC4* are highly expressed in m6A methylation subtype B and m6A genotype B. (B) *HDAC1*, *HDAC3* and *HDAC6* are highly expressed in m6A methylation subtypes A and m6A genotype A.

The expression profiles of HDACs revealed notable differences across m6A methylation subtypes. HDAC2 and HDAC4 were predominantly expressed in m6A methylation subtype B and genotype B, while HDAC1, HDAC3 and HDAC6 showed higher expression levels in m6A methylation subtype A and genotype A (Figure 9A,B).

### 3.10 Expression of HDAC2 in BEAS‐2B Cells by Immunofluorescence

**FIGURE 10:**
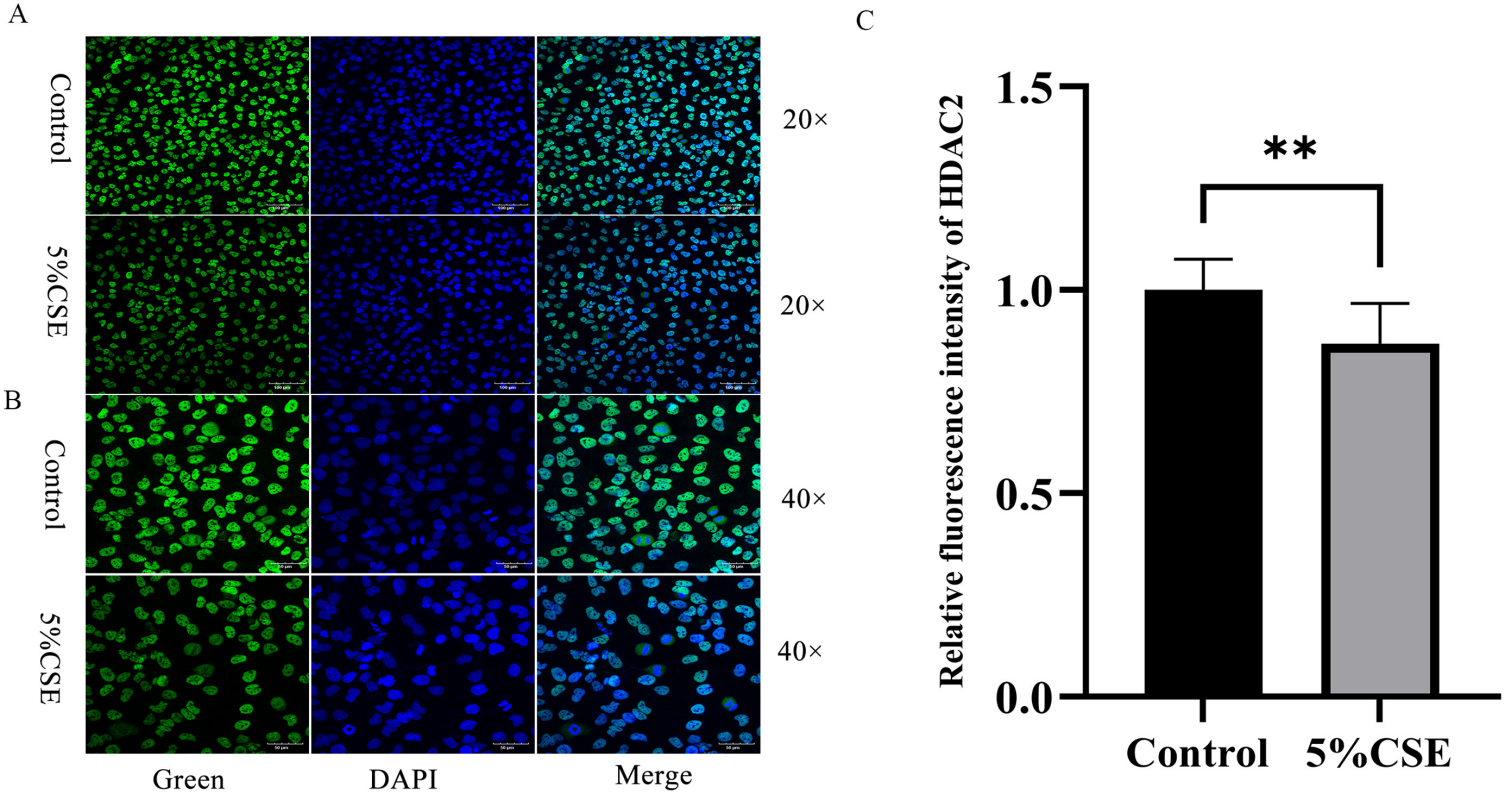
Expression result of *HDAC2* in immunocyte fluorescence. In control, *HDAC2* was highly expressed under 20 times and 40 times fluorescence microscope; In 5% CSE group, *HDAC2* was low expressed under 20 times and 40 times fluorescence microscope. (A) HDAC2 in 20× (B) HDAC2 in 40× (C) Fluorescence expressions of HDAC2. **p < 0.01.

Immunofluorescence staining demonstrated that BEAS‐2b cells in the control group maintained a normal, spindle‐shaped morphology under 20x and 40x magnification. These cells exhibited intact cell membranes and nuclei with prominent HDAC2 expression in the nucleus (Figure 10A–C). In contrast, exposure to 5% CSE disrupted the normal cellular architecture, causing the cells to become rounded and leading to membrane and nuclear degradation. Concurrently, HDAC2 expression was significantly reduced, indicating that CSE exposure can diminish HDAC2 levels and disrupt cell morphology (Figure 10A–C).

### 3.11 Western Blotting of METTL3, NF‐kB and HDAC2

**FIGURE 11:**
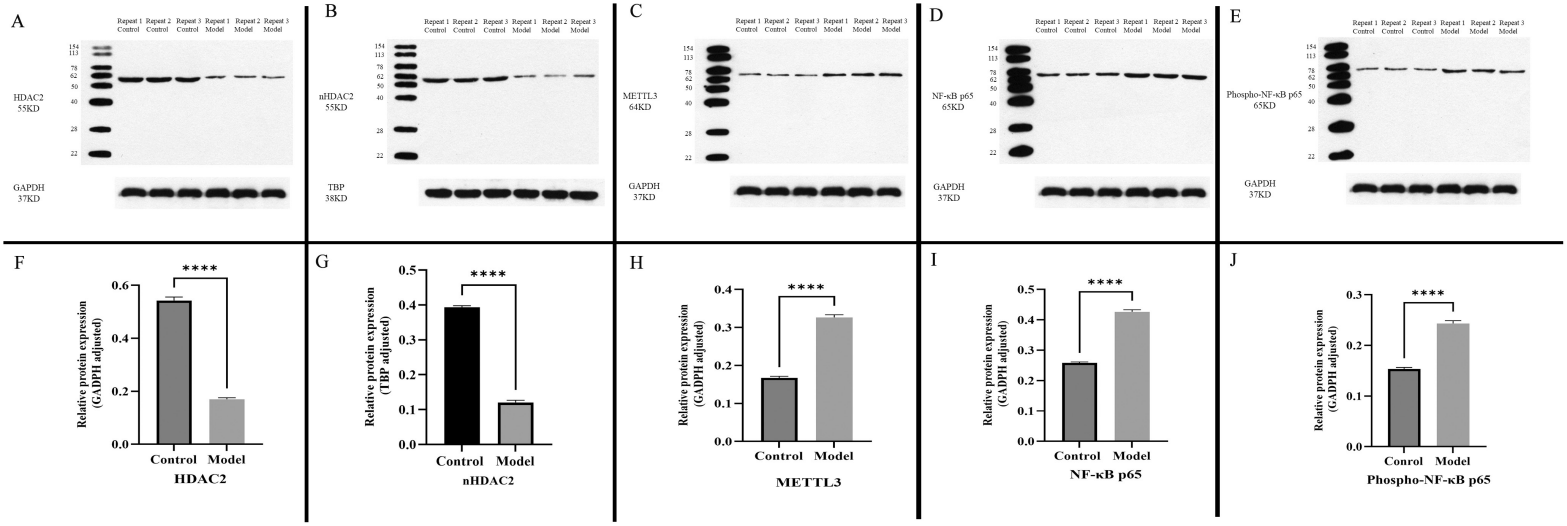
Western blotting (A) *HDAC2* (B) *nHDAC2* (C) *METTL3* (D) *NF‐kB* (E) *Phospho‐NF‐kB*. (F) Histogram of *HDAC2* protein expression in the control and 5% CSE groups. (G) Histogram of *nHDAC2* protein expression in the control and 5% CSE groups. (H) Histogram of *METTL3* protein expression in the control and 5% CSE groups. (I) The Histogram of *NF‐kB* protein expression in the control and 5% CSE groups. (J) Histogram of *Phospho‐NF‐kB* protein expression in the control and 5% CSE groups. ****p < 0.000.

Western blotting was used to detect the expression of various proteins in BEAS‐2B cells. CSE induced significantly higher expression of METTL3, NF‐kB and phospho‐NF‐kB in the 5% CSE model group than in the control group, while HDAC2 and nuclear HDAC2 (nHDAC2) expression was significantly reduced. This suggests that CSE may trigger inflammatory responses through the activation of METTL3 and NF‐κB pathways, which, in turn, could suppress HDAC2 expression (*p* values: METTL3 < 0.000, NF‐kB < 0.000, nNF‐kB < 0.000, HDAC2 < 0.000, phospho‐NF‐kB < 0.000, Figure 11A–J).

## 4 Discussion

In this study, we constructed an RF model and nomogram model by m6A DEMs to predict the incidence and validity rate of COPD. Furthermore, we classified COPD into two subtypes based on the m6A DEMs. We found that cellular immunity involved eosinophils and neutrophils, which were highly enriched in Cluster B, and monocytes and activated B cells, which were highly enriched in Cluster A.

### 4.1 Twenty‐Three m6A Methylation Regulators With Differential Expression in COPD

Differential analysis of 23 m6A methylation regulators highlighted that several, including *METTL3*, *RBM15*, *RNM15B*, *CBLL1*, *YTHDC2*, *YTHDF1*, *YTHDF2*, *HNNRPC*, *LPPPRC*, *RBMX*, *FTO* and *ALKBH5*, were significantly overexpressed in COPD patients. These regulators promote various biological processes associated with COPD development [23]. For instance, overexpression of *METTL3* has been linked to enhanced inflammatory responses through the *TRAF6*, NF‐KB and MAPK signalling pathways, while inhibition of *METTL3* reduces IL‐1β‐induced inflammation [24]. As a demethylase, FTO plays a role in modulating inflammatory responses by affecting the chemotaxis of interferon‐stimulating genes and proinflammatory factors such as *TNF*, *IL6*, *IL9*, *IL10* and *IL11* [25].

### 4.2 Establishment of COPD Prediction Model Based on DEMs

Using an RF model, SVM model and nomogram, the study identified 11 m6A methylation regulators (*LRPPRC*, *YTHDC1*, *YTHDF1*, *HNRNPA2B1*, *WTAP*, *CBLL1*, *RBM15*, *HNRNPC*, *YTHDF2*, *RNMX* and *FTO*) that could predict the occurrence of COPD effectively. Through the validation of the DCA curve, we found that this model is beneficial to COPD patients. Mitochondrial‐related functional protein (*LRPPRC*) can regulate mitochondrial gene expression and function, cell cycle progression and tumourigenesis, and the overexpression of *LRPPRC* is related to the growth of gastric cancer, while inhibiting the expression of *LRPPRC* can inhibit the proliferation of gastric cancer cells [26]. Further studies have shown that knockout of the *LRPPRC* gene in mice can damage the assembly and enzyme activity of ATP synthase to induce mitochondrial respiratory dysfunction and increase the production of reactive oxygen species [27]. *YTHDC1* is an m6A methylation regulator that can regulate the nuclear output of mRNA and the nuclear stability of RNA by regulating the precursor of mRNA. Studies have shown that *YTHDC1* can reduce the inflammatory response by stabilising deacetylase 1 (*SIRT1*) mRNA to reduce *STAT3* acetylation.

Further studies have shown that knocking down the expression of *YTHDC1* in mouse microglia (BV2) cell lines can promote the expression of proinflammatory phenotypic markers and proinflammatory cytokines and promote the migration of microglia [28]. Wilms' tumour‐associated protein (*WTAP*) can affect mRNA modification to regulate gene expression. Studies have found that *WTAP* can regulate the expression of mRNA that activates transcription factor 4 (*AFT4*) to promote the endoplasmic reticulum stress response and apoptosis, thereby promoting myocardial ischemia/reperfusion injury, while knocking down the expression of *WTAP* can inhibit endoplasmic reticulum stress and myocardial ischemia/reperfusion injury in cardiomyocytes (AC16) [29]. RNA binding motif protein 15 (*RBM15*) is involved in the methyltransferase complex and has been shown to impact the growth of various tumours, notably clear cell renal cell carcinoma. The influence of RBM15 may be mediated via the EP300/CBP‐RBM15‐CXCL11 axis, highlighting its potential role in tumour biology [30]. Heteronuclear ribonucleoprotein C (*HNRNPC*) affects mRNA stability, output and translation by binding to RNA transcripts. Research suggests that adriamycin‐related lncRNA *SNHG1* can interact with nucleolar proteins, remaining in the nucleus and competitively binding to *HNRNPC* alongside p53. This interaction diminishes *HNRNPC*'s regulatory effect on p53 activity, thereby upregulating p53 and promoting p53‐dependent apoptosis [31].

### 4.3 GO and KEGG Enrichment Analysis of m6A Genotyping Differential Genes

COPD is recognised for its heterogeneity, manifested through different inflammatory profiles. The inflammatory landscape in COPD includes type 1 inflammation characterised by increased neutrophils, alveolar macrophages and CD8+T lymphocytes, and type 2 inflammation marked by elevated eosinophils [32, 33, 34]. Differences in the expression levels of cytokines such as IL‐1β, TNF‐α and IL‐17 have been noted among these subgroups [2].

Our previous studies employing consensus clustering have identified distinct clinical subgroups of COPD, each with unique gene expression patterns [35]. Enrichment analyses of DEGs from these subgroups have highlighted key biological functions such as helicase activity regulation, response to interleukin‐1, histone deacetylase binding and CXCR chemokine receptor binding. Similarly, KEGG pathways enriched among these DEGs include the NF‐κB signalling pathway, tumour necrosis factor signalling pathway and *IL‐17* signalling pathway.

*IL‐1β*, a cytokine released by various inflammatory cells (neutrophils, macrophages, eosinophils), plays a crucial role in COPD by activating cell nuclear factors and the NF‐KB pathway, leading to alveolar septal destruction and airway wall fibrosis [36]. Cigarette smoke, a common inducer of chronic COPD in experimental models, has been shown to diminish histone deacetylase activity, thereby enhancing *IL‐8* and matrix metalloproteinase expression and perpetuating inflammation [37]. In respiratory diseases like asthma and COPD, NF‐κB, a central inflammatory mediator, can be activated by cytokines such as IL‐1β and TNF‐α. This activation promotes inflammatory cell infiltration, apoptosis and oxidative stress within the pulmonary environment [38]. Moreover, *TNF‐*α can trigger the TNF‐α/NF‐KB signalling pathway through TNFR1 and TRAF2, releasing IL‐8 [39].

Analysis of immune cell infiltration has found that cigarette smoke can promote the expression of *IL‐1*, *TNF* and other inflammatory factors by activating the NF‐KB pathway in human BEAS‐2B and human bronchial epithelial (HBE) cells, leading to inflammation, apoptosis and oxidative stress in COPD. *TNF‐*α can activate the TNF‐α/NF KB signalling pathway to induce the release of interleukin‐8 (IL‐8) by activating tumour necrosis factor receptor 1 (*TNFR1*) and recruiting tumour necrosis factor receptor–related factor 2 (*TRAF2*) in the COPD response.

### 4.4 Immune Cell Infiltration in m6A Methylation Subtypes and m6A Genotype

Through the analysis of immune cell infiltration, we found that compared with the groups with high expression of *WTAP*, the group with low expression of *WTAP* had significantly higher levels of neutrophils and eosinophils. This suggests a link between m6A methylation subtypes and specific extracellular traps: neutrophil extracellular traps (NETs) and eosinophil extracellular traps (EETs) in Cluster B, and monocyte extracellular traps (MOETs) in Cluster A.

EETs comprise DNA fibre networks released by activated eosinophils, including granular proteases such as eosinophil cationic protein and major basic protein. Activated eosinophils can induce steroid resistance in severe asthma by activating type 2 innate immune lymphocytes through EETs [40].

NETs are reticular extracellular structures composed of extracellular DNA, neutrophil elastase (NE), myeloperoxidase (MPO) and other degradative enzymes released by activated neutrophils. In COPD, enhanced NET formation is associated with airflow restriction and lung tissue damage by promoting airway neutrophil inflammation [41, 42]. Hypoxia alongside inflammatory mediators can further induce the release of tissue‐damaging proteins such as NE and MPO, exacerbating endothelial injury in COPD [43].

MOETs, activated by reactive oxygen species (ROS), share morphological similarities with NETs, including the release of myeloperoxidase (MPO), lactoferrin (LF) and elastase. However, the release of MOET does not depend on MPO activity. Other studies have found that hypoxia can cooperate with inflammatory mediators to promote the release of tissue toxic proteins such as NE, myeloperoxidase (MPO), vascular injury or activated intercellular adhesion molecule (ICAM‐1) in COPD to induce endothelial injury. Other studies have shown that C5AR1‐positive neutrophils can also release inflammatory factors such as *IL1* β, *TNF α* and *ENO1*, which are known to promote tumour progression in breast cancer. However, m6A‐methylated *WTAP* can prevent this process [44]. This regulatory mechanism highlights the potential of m6A methylation as a therapeutic target in inflammation‐driven diseases.

The leukocyte chemokine receptor CXCR1 in COPD patients, by binding with its ligand *IL‐8*, can activate neutrophil chemotaxis, leading to enhanced phospholipase D activation and respiratory bursts. This interaction promotes airflow restriction and amplifies the inflammatory response in COPD [45].

Increased eosinophil counts in the blood and sputum of COPD patients can predict the risk of disease exacerbation and the response to glucocorticoid treatment, indicating a significant biomarker for managing COPD [46]. Furthermore, a rise in blood eosinophils correlates with better lung function and a higher FeNO value, emphasising their role in lung physiology and disease progression [47].

### 4.5 HDAC Family Protein Expression in m6A Methylation Subtypes and m6A Genotype

By comparing m6A methylation typing and m6A genotyping with the box diagram of HDAC family protein expression, we found that HDAC2 and HDAC4 were expressed at low levels in m6A methylation type A and m6A genotype A, while *HDAC1, HDAC3* and *HDAC6* were expressed at low levels in m6A methylation genotype B and m6A genotype B. Ergosterol can improve the activity of *HDAC3* in macrophages and inhibit the activation of PCAF, as well as acetylate NF‐KB/p65 to intervene in the inflammatory response in COPD [48]. *HDAC6* is a cytoplasmic deacetylase that can regulate various cellular processes. *HDAC6* can activate the expression of actin (ERK) in the airway and induce pulmonary vascular remodelling in COPD, while *HDAC6* inhibitors can significantly inhibit airway remodelling and increase airway resistance induced by cigarette smoke (CSE) in COPD rats [49]. The decrease in *HDAC2* expression in COPD patients can induce the expression of *IL‐17A* and airway remodelling. The activator of *HDAC2* can prevent airway remodelling by inhibiting airway inflammation [50]. NETs play a central role in pathogen containment and immune‐mediated inflammatory diseases, and studies have found that the class I *HDAC* (*HDAC1/2/3/8*) and class IIB *HDAC* (*HDAC6/10*) inhibitor ricolinostat can inhibit the production of NETs and proinflammatory cells by inhibiting the release of citrullinated histone H3 (cith3) to reduce the incidence rate of pneumonia and septic shock in mice and further improve lung function [51]. Further studies have shown that histone deacetylase inhibitors (HDACIs) can increase histone acetylation and inhibit the formation of NETs in a dose‐dependent manner [52].

### 4.6 Experimental Verification

METTL3, a pivotal catalytic enzyme, plays a crucial role in m6A methylation, influencing the proliferation, migration and drug resistance of tumour cells. The knockdown of METTL3 significantly diminishes m6A methylation transcription across various cells [53]. Notably, METTL3 enhances the development of lung adenocarcinoma, likely through the promotion of tumour occurrence and the inhibition of cell iron death by upregulating SLC7A11 expression [54]. However, the specific mechanisms through which METTL3 contributes to the development of COPD remain unclear. To explore this, we utilised CSE to induce COPD in BEAS‐2B cells, creating a model to investigate METTL3, NF‐kB and HDAC2 interactions in COPD [38]. Immunocytochemical fluorescence assays demonstrated that 5% CSE impairs the activity and morphology of BEAS‐2B cells and reduces nuclear HDAC2 expression. Western blot analysis confirmed that, compared to the control group, the 5% CSE model group exhibited significantly higher levels of METTL3 and NF‐kB, while HDAC2 expression was markedly reduced. Additionally, stimulation of HBE cells with CSE has been shown to elevate METTL3 levels and enhance E‐cadherin expression and epithelial–mesenchymal transition (EMT), potentially fostering lung cancer development [55]. Furthermore, in a microglial inflammation model induced by LPS, METTL3 was found to promote the release of proinflammatory cytokines such as IL‐1β, IL‐6, TNF‐α and IL‐18 via the TRAF6/NF‐kB pathway [55]. Research indicates that Anemoside B4 improves lung function and reduces inflammatory infiltration in COPD mice, possibly by decreasing myeloperoxidase (MPO) levels and ROS production in CSE‐induced human bronchial epithelial cells (HBC) [56]. Moreover, CSE stimulation significantly increases extracellular DNA levels and the activity of NE and MPO associated with NETs [57]. CSE also activates NF‐kB, leading to the induction of various proinflammatory factors, chemokines, enzymes and adhesion molecules, with the transcription of these factors requiring histone acetylation during COPD progression [58, 59]. Additionally, exposure to CSE notably decreases HDAC2 expression and upregulates NF‐kB in rats and HBE cells, suggesting a potential mechanism whereby CSE‐induced airway inflammation might suppress HDAC2 expression and elevate NF‐kB levels [60].

### 4.7 Limitations of This Study

Research indicates that the m6A methylation factor significantly influences gene expression regulation in COPD [21]. In COPD animal models, distinct expressions of m6A regulatory factors are observed between acute exacerbation and stable phases. Pearson's correlation analysis revealed a positive correlation between the methylation levels of these DEGs and their expression levels during both stable and acute exacerbation periods [21]. This suggests that m6A methylation is crucial for maintaining mRNA stability in COPD. Enrichment analysis identified that these differentially methylated genes are linked to vital biological processes, including inflammation, immune system processes, IL‐17 signalling pathway and TNF signalling pathway [21], highlighting their roles in the immune responses during different COPD phases. Due to certain limitations, this study could not delve deeper into these findings, indicating a need for future research.

A comprehensive analysis of 15 critical m6A regulators revealed that m6A methylation subtypes A and B (Clusters A and B) are associated with neutrophil, eosinophil and monocyte traps. Further analysis of the functional pathways enriched by m6A methylation typing (DEGs) and the interaction between different methylation types and the HDAC protein revealed that the m6A methylation regulator, METTL3, might enhance the inflammatory response, airway remodelling, and airway resistance in COPD through regulatory mechanisms involving NF‐kB and HDAC protein expression. Subsequent cellular experiments confirmed that METTL3 plays a significant role in COPD pathogenesis by modulating the NF‐kB‐MPO pathway, leading to reduced HDAC2 expression. These findings suggest that targeting METTL3 and other m6A methylation regulators could offer novel therapeutic avenues for COPD treatment.

## 5 Conclusions

This study underscores the potential of m6A methylation regulators in aiding the diagnosis and subtype classification of COPD, offering new insights into the molecular mechanisms underpinning this complex disease.

## Author Contributions

**Pingan Zhang:** conceptualization (equal), data curation (equal), formal analysis (equal), investigation (equal), resources (equal), visualization (equal), writing – original draft (equal). **Na Gao:** conceptualization (equal), data curation (equal), investigation (equal), resources (equal), visualization (equal), writing – original draft (equal). **Xiaoning Li:** data curation (equal), investigation (equal), resources (equal), writing – original draft (equal). **Xudong Zheng:** investigation (equal). **Deyu Kong:** investigation (equal). **Jianjun Wu:** conceptualization (equal), resources (equal), visualization (equal), writing – original draft (equal), writing – review and editing (lead).

## Conflicts of Interest

The authors declare no conflicts of interest.

## Data Availability

The data supporting the findings of this study are available within the article.
